# 0595. Quantitating granulocyte reactive oxygen species production by flow cytometry in a clinical setting

**DOI:** 10.1186/2197-425X-2-S1-P38

**Published:** 2014-09-26

**Authors:** B Smit, MC de Waard, YM Smulders, HM Oudemans-van Straaten, C Boer, AB Vonk, D Veerhoek, JJ García Vallejo, S Kamminga, AR Girbes, AM Spoelstra-de Man

**Affiliations:** VU University Medical Center, Intensive Care, Amsterdam, Netherlands; VU University Medical Center, Internal Medicine, Amsterdam, Netherlands; VU University Medical Center, Anaesthesiology, Amsterdam, Netherlands; VU University Medical Center, Cardiothoracal Surgery, Amsterdam, Netherlands; VU University Medical Center, Clinical Perfusion, Amsterdam, Netherlands; VUmc, Molecular Cell Biology & Immunology, Amsterdam, Netherlands; VU University Medical Center, Amsterdam, Netherlands

## Introduction

Oxidative stress is an important part of a wide range of pathologies and therefore an interesting parameter to determine. However, the detection of reactive oxygen species (ROS), is not straightforward. Sample heterogeneity, delayed analysis and sample preparation, reduces specificity and sensitivity due to probe activation by light, air, probe leakage from cells or cellular activation by sample manipulation. Hence, samples should be processed minimally and analysed immediately if possible. Since clinical research can be subject to uncontrollable timetables and only few departments have access to a dedicated laboratory, the quantification of ROS is challenging.

## Objectives

For the purpose of a randomized-controlled trial involving Coronary Artery Bypass Graft surgery (CABG), we sought to establish a flow cytometric assay to detect ROS production in granulocytes (PMN) that is easy to perform, does not require a specialised laboratory on-site and is relatively insensitive to delay between sample preparation and analysis. The fluorescent probe applied is CellROX® Green (CrX; Life Technologies, Eugene, Oregon, USA), which becomes fluorescent after oxidation and subsequently binds to DNA.

## Methods

Whole blood samples were collected from healthy volunteers and from patients before (baseline) and during CABG surgery (after 1 hour of cardiopulmonary bypass, CPB). 30 µl whole blood was incubated for 1 hour with 10µM CrX immediately after sampling. To compensate for the surgery induced PMN count increase, 1 hour CPB samples were incubated with 10, 20 and 30µM CrX. Following incubation, erythrocytes were lysed by hypotonic shock and the sample was washed three times with Phosphate Buffered Saline (PBS). Finally, leukocytes were stored in 200µl of PBS at room temperature, in the dark, until analysis. Positive and negative controls were obtained by additional incubation with phorbol-12-myristate-13-acetate and N-acetyl-cysteine. Mean Fluorescence Intensity (MFI) of the PMN population, which is proportional to the ROS produced, was measured in the FL1 channel of a BD FACSCalibur as soon as possible after incubation of the second CABG sample (~5 hours). PMN were identified based on their forward and sideward scatter characteristics to avoid fluorescence compensation corrections and aspecific activation of neutrophils by fluorescent cell-specific antibodies.

## Results

**Figure 1:**
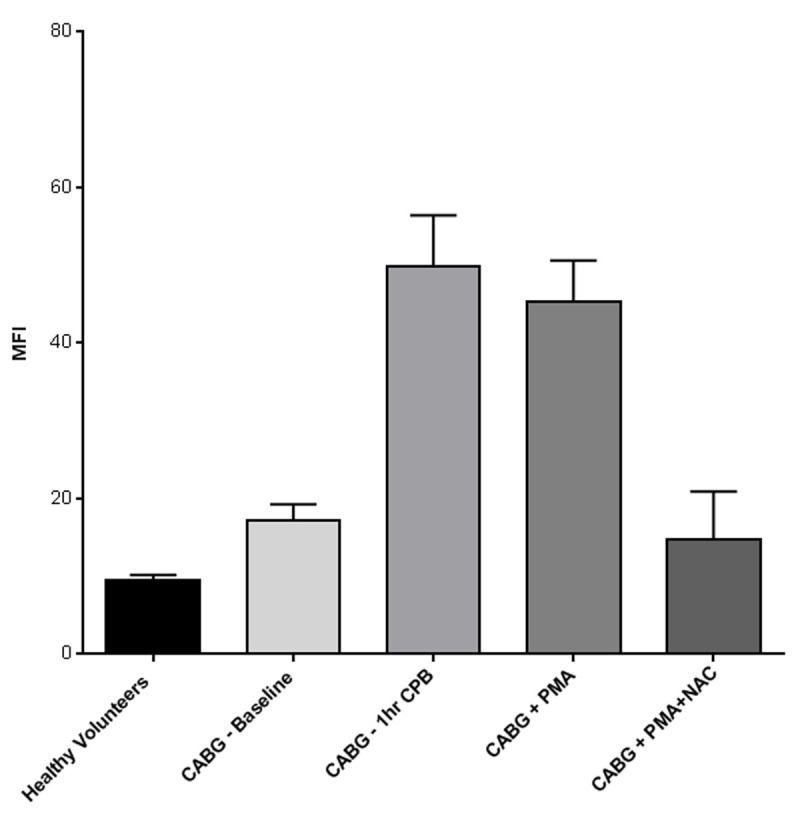
Figure 1

In line with our expectations, little production of ROS was demonstrated in healthy volunteers (n=4) and baseline samples from CABG surgery patients, while a marked increase was found after one hour of CPB (n=8, figure 1). PMA induces a NAC-inhibitable increase of MFI in CABG baseline samples. Interestingly, we were also able to detect what appears as separate ROS producing neutrophil populations, which warrants further investigation.

## Conclusions

Our current method is easy to perform, implementable into clinical research and is able to detect different ROS responses.

